# Characterization of *Spodoptera litura* Gut Bacteria and Their Role in Feeding and Growth of the Host

**DOI:** 10.3389/fmicb.2020.01492

**Published:** 2020-06-30

**Authors:** Xiaofeng Xia, Bomiao Lan, Xinping Tao, Junhan Lin, Minsheng You

**Affiliations:** ^1^State Key Laboratory of Ecological Pest Control for Fujian and Taiwan Crops, Institute of Applied Ecology, Fujian Agriculture and Forestry University, Fuzhou, China; ^2^Joint International Research Laboratory of Ecological Pest Control, Ministry of Education, Fuzhou, China; ^3^Key Laboratory of Integrated Pest Management for Fujian-Taiwan Crops, Ministry of Agriculture, Fuzhou, China; ^4^Quanzhou Institute of Agricultural Sciences, Quanzhou, China; ^5^Fujian Vocational College of Bioengineering, Fuzhou, China

**Keywords:** detoxification, food utilization, gut bacteria, metagenomic, nutrient supply, *Spodoptera litura*

## Abstract

Insect gut microbes play important roles in host feeding, digestion, immunity, growth and development. *Spodoptera litura* is an important agricultural pest distributed of global importance. In the present study, diversity and functions of the gut bacteria in *S. litura* are investigated based on the approaches of metagenomics and denaturing gradient gel electrophoresis (DGGE). The results showed that the gut bacterial diversity of *S. litura* reared on taro leaves or an artificial diet, were similar at the phylum level, as both were mainly composed of Proteobacteria, but differed significantly at the order level. *Spodoptera litura* reared on taro leaves (Sl-tar) had gut biota mainly comprised of Enterobacteriales and Lactobacillales, while those reared on artificial diet (Sl-art) predominantly contained Pseudomonadales and Enterobacteriales, suggesting that gut bacteria composition was closely related to the insect’s diet. We found that feeding and growth of *S. litura* were significantly reduced when individuals were treated with antibiotics, but could be both restored to a certain extent after reimporting gut bacteria, indicating that gut bacteria are important for feeding, digestion, and utilization of food in *S. litura*. Metagenomic sequencing of gut microbes revealed that the gut bacteria encode a large number of enzymes involved in digestion, detoxification, and nutrient supply, implying that the gut microbes may be essential for improving the efficiency of food utilization in *S. litura*.

## Introduction

The insect gut hosts a large number of microbes, many of which play an important role for the insect in feeding, digestion, immunity, growth, development, and even insecticide resistance. These functions are achieved through variety of enzymes they produce that can, among other things, promote the degradation of toxic substances, synthesis of amino acids, metabolization, and utilization of carbohydrates (Hansen and Moran, 2011; Kikuchi et al., 2012; Engel and Moran, 2013; Chomwong et al., 2018; Xia et al., 2018).

It has been shown that symbiotic gut microbes of *Plutella xylostella* can degrade secondary metabolites such as phenol (Xia et al., 2017), and *Acinetobacter* spp. in the gypsy moth, *Lymantria dispar*, gut degrade toxic phenolic compounds produced by aspen, thus protecting its host from harm after eating the foliage (Mason et al., 2016). Toxic substances such as secondary metabolites, *Pseudomonas fulva*, isolated from the gut tract of the coffee berry borer, *Hypothenemus hampei*, were shown to degrade caffeine and help the host adapt to consumption of coffee beans (Ceja-Navarro et al., 2015). Amino acids are essential nutrients for the growth and development of organisms. Many animals cannot synthesize essential amino acids by themselves and require supplement through their diet (Kasting and McGinnis, 1958). Symbiotic bacteria of some insects can synthesize essential amino acids for their hosts, or synthesize essential amino acids together with the host, which is most common in true bugs (Sasaki and Ishikawa, 1995; Hansen and Moran, 2011). Gut microbes have the potential to provide essential amino acids for their hosts, either as they are secreted outside the cell for host use or their bacterial residues are available to be reused as nutrients (Engel and Moran, 2013; Xia et al., 2017). The food of phytophagous insects is rich in cellulose and other polysaccharide compounds, but the host insects often do not possess the degradation enzymes of these substances. Gut microbes usually play an important role in the digestion and metabolism of the polysaccharide (Engel and Moran, 2013). A typical example of this symbiotic relationship is the gut microbes of termites in the genus *Nasutitermes* which help the host to degrade lignocellulose (Watanabe and Tokuda, 2010). Other studies in *P. xylostella* and *Bombyx mori* revealed that their gut microbes are active in the digestion of cellulose, xylan and pectin (Anand et al., 2010; Xia et al., 2017). Similarly in honeybees, *Apis mellifera*, microbes play a role in degrading pectin found in pollen walls, permitting digestion by the bees (Engel et al., 2012).

The diversity of gut microbes in insects determines their host functions. This diversity is affected by many factors, including the structure of the gut tract and environmental factors such as gut pH, redox potential, the presence of digestive enzymes and the food they consume (Yun et al., 2014). Previous studies have shown that diet has a considerable impact on the gut microbial diversity of insects. When studying the effect of diet on *Helicoverpa armigera* larvae, Priya et al. (2012) found that diversity of the gut microbiota was greatly increased when it fed on different foods than when it lived in different areas but maintained the same diet. These results demonstrated the role that host plants have on the gut bacteria of *H. armigera*. Further studies have shown that the gut bacteria of *H. armigera* are similar to the microbial communities in the leaves it feeds on, suggesting that the gut bacteria of *H. armigera* are influenced by the symbiotic bacteria on the surface of the leaves (Priya et al., 2012). A study of mosquitoes has shown that food changes during the adult stage significantly affect the composition of gut symbiotic bacteria (Wang et al., 2011). As in *Spodoptera littoralis*, the diversity of gut bacteria was significantly different when it was reared on different plants, Lima bean vs. barley, and the gut bacteria of *H. armigera* were also different between groups reared on cabbage, cotton or tomato (Tang et al., 2012). A more comprehensive study compared 62 different insect species and found that gut bacteria could be altered when insects ingested foods containing lignocellulose-derived substances (Colman et al., 2012).

The tobacco cutworm, *Spodoptera litura* (Fabr.) (Lepidoptera: Noctuidae), is global agricultural pest. It is highly polyphagous, able to feed on many families of plants, including important crops such as cotton, beans, tobacco, vegetables, and rice (Dhir et al., 1992; Qin et al., 2004; Zhou, 2009; Ahmad et al., 2013). Previous studies have begun to explore gut microbial diversity and function of *S. litura*. Bapatla et al. (2018) used the V3 region of 16S rRNA to amplify the gut bacteria in *S. litura* and then sequenced them on the Illumina MiSeq platform, revealing that the gut bacteria of *S. litura* were composed of 27% Proteobacteria and 14% Chlorobi. Proteobacteria was the most abundant phylum in the *S. litura* gut, however, 34% of unclassified bacteria in the V3 region were relatively short and difficult to annotate (Bapatla et al., 2018). Although there have been some studies on the gut microbes of *S. litura*, there is still a lack of systematic studies on the diversity of gut bacteria and their interaction with food. In this study, three questions were posed: (1) What is the diversity of gut microbes of *S. litura* and their relationship with food? (2) Do the gut bacteria promote the feeding and growth of *S. litura*? (3) What are the potential functions of gut microbes of *S. litura*, especially in food metabolism and digestion? Based on these, metagenomic sequencing and DGGE techniques were used to investigate the effects of food on the gut bacterial diversity of *S. litura*. The role of gut bacteria in feeding and growth of *S. litura* was analyzed by clearance and reintroduction of the bacteria. The functions of gut bacteria in food digestion, nutrition supply and detoxification were revealed by using metagenomic data. The analysis of the potential mechanisms that mediate the changes in food-use efficiency lays the foundation for further study of gut bacterial in *S. litura*.

## Materials and Methods

### Insect Rearing

*Spodoptera litura* were collected from the taro planting base in Nantong, Fuzhou (26.08°N, 119.28°E). These were split into two groups with one reared for more than five generations on taro leaves (Sl-tar) in the laboratory at 24 ± 2°C, 60%–80% RH, and 16L:8D and the other reared on an artificial diet (Sl-art) for the same duration and under the same conditions. The formula for the artificial diet was 100 g of soybean powder, 80 g of wheat bran, 26 g of yeast powder, 8 g of casein, 8 g of vitamin C, 1 g of choline chloride, 2 g of sorbic acid, 0.2 g of cholesterol, 0.2 g of inositol, 26 g of agar, and 1000 mL of distilled water.

### Antibiotic Application

To study the effect of antibiotics on the bacterial diversity in the gut of *S. litura*, the taro leaves fed to the insects were first soaked with antibiotics. A solution of 1 mg/mL ciprofloxacin, 1 mg/mL levofloxacin and 2 mg/mL metronidazole were prepared. The taro leaves were soaked in this solution for 20 min, and 1% Tween-20 surfactant was added to enhance the adsorption capacity of antibiotics on taro leaves. Fifty 4th instar larvae were taken from the colony and reared in plastic boxes for 2 days with the treated leaves as their diet. The treated leaves were changed once a day. A control group was prepared in the same manner with the taro leaves soaked in sterile water only. All treatments were repeated three times.

For those fed on artificial diet, a mixture of 1 mg/mL ciprofloxacin, 1 mg/mL levofloxacin and 2 mg/mL metronidazole artificial diet was prepared, and provided to the *S. litura*. The artificial diet without antibiotic was used as a control. All treatments were repeated three times.

### Metagenomic DNA Extraction of the *S. litura* Gut Microbiota

Twenty, healthy 4th instar larvae of *S. litura* (Sl-tar, Sl-art) were randomly selected and starved for 24 h before dissection. The dissection tools were sterilized by 75% alcohol and irradiated for 30 min by UV. Moreover, 75% alcohol was used to sterilize the body surface of *S. litura* for 20 s, which was then rinsed in distilled water. The larvae were dissected under aseptic conditions, and the gut contents were mixed in 2 mL sterile water. The QIAamp DNA Stool Mini Kit (Qiagen, United States) was used to extract the total metagenomic DNA from the gut content. Extracted DNA integrity was confirmed by agarose gel electrophoresis. Total DNA was sent to Beijing Novogene Bioinformatics Technology Co., Ltd. for sequencing.

### Metagenomic Sequencing and Data Analysis of the *S. litura* Gut Microbiota

A 300 bp paired-end library was constructed and the metagenomic DNA isolated from both Sl-tar and Sl-art gut content were sequenced by the PE125 protocol on the Illumina HiSeq 2500. The raw data were processed as follows to obtain the clean data: (1) Removal of the reads with low-quality bases (*Q* ≤ 5) exceeding a certain proportion (40% set by default reads), (2) removal of the reads containing >10% “N” bases, and (3) removal of the reads with >15 bp overlap with the adapter. The clean data were then analyzed using SOAP *de novo* (Version 2.21) assembly software to perform assembly analysis (Luo et al., 2012). First, we selected different K-mers (default selection 49, 55, 59) to assemble, for further analysis, the assembly with the largest N50 metrics was selected. The assembled scaffolds were interrupted from the N junction to obtain contigs without N. CD-HIT software (Version: 4.5.8) was used to remove all redundant contigs (Default value of the sequences consistency was 95%) (Li and Godzik, 2006). As the assembly quality of the Sl-tar was not well as Sl-art, which may be affected by its food of plant residue. The plant residue may affect the DNA extraction, amplification and the library construction, which make a result of low assembly quality. Thus, in order to improve the annotation rate of each sample, we mixed the contigs of the two samples together, and the clean data of each sample was mapped to the non-redundant contigs by SoapAligner (Version: 2.21) to get the relative abundance of each contigs. The contigs with the depths of 0 were filtered out. The non-redundant contigs were BLASTed (*E*-value ≤ 1e-5) against Bacteria, Fungi, Archaea, and Viruses in the NCBI NT database (NT Version: 2014-10-19). The LCA algorithm in MEGAN 4 was used to annotate the species information (Huson et al., 2011), and the relative abundance information at different taxonomic levels was obtained based on the results of LCA annotation and contig abundance. The annotation results of species profiling were visualized by Krona (Ondov et al., 2011). A length of contigs ≥300 bp was selected, and MetaGeneMark software (Version 2.10) was used to identify gene information for functional annotation (Zhu et al., 2010). The abundance of each gene in the samples was calculated as follows:

$$a\,b\,u\,n\,d\,a\,n\,c\,e\,\left(g\right)=\sum_{r\in R}\frac{b\,a\,s\,e\,\_\,o\,v\,e\,r\,l\,a\,p\,\left(g,r\right)}{b\,a\,s\,e\,\_\,l\,e\,n\,g\,t\,h\,\left(g\right)}$$

(In sample *i*, the length that reads compare to gene *g* is *R*, where base_overlap refers to the depth at a certain site of gene *g*, base_length is the length of gene *g*) (Arumugam et al., 2011).

The redundant sequences were BLASTed against three functional databases [Kyoto Encyclopedia of Genes and Genomes (KEGG, Version: 58), Evolutionary Genealogy of Genes: Nonsupervised Orthologous Groups (EggNOG, Version: 4.0), Carbohydrate-Active enzymes Database (CAZy, Version: 2014.11.25)] to predict the functions of the gene. The lowest Blast E-value was selected from the comparison results of each sequence. At the same time, the BCR (the blast coverage ratio) of each gene between reference (Ref.) and query (que.) was calculated to ensure that the BCR (Ref.) and BCR (que.) were both greater than 40%. In order to analyze the relationship between the glycoside hydrolases (GHs) richness and food complexity in different insect’s gut microbes. The GHs gene families of *S. litura* were compared with that of other published species, such as *P. xylostella* (Xia et al., 2017), *Bos grunniens* (Dai et al., 2012), *Nasutitermes exitiou* (Warnecke et al., 2007), and *Apis mellifera* (Engel et al., 2012). The raw sequences of the *S. litura* gut microbiota were deposited in the Sequence Read Archive (SRA) database under accession number SRP116674.

### PCR-DGGE Analysis of Bacterial Diversity in the Gut Tract of *S. litura*

*S. litura* were collected side-by-side from the same batch of insects, the method of gut content collection from *S. litura* larvae was the same as previously described, except the PowerSoil^®^DNA Isolation Kit (MO BIO Laboratories, San Diego Biotech Corridor, United States) was used for total DNA isolation. The extracted total DNA of gut bacteria was used as a template, and universal primers for the bacterial 16S rDNA V3 region, 343F (5′-ACT CCTACGGGAGGCAGCAG-3′) and 534R (5′-ATTACCGCGGCTGCTGG-3′), were used for DNA amplification. The 5′ end of the forward primer was appended with a 40 bp GC-clip (CGCCCGCCGCGCGCGGCG GGCGGGGCGGGGGCACGGGGGG) (Muyzer et al., 1993; Nakatsu et al., 2000). The PCR mix was 25 μL, and the drop PCR procedure was used. The PCR program was as follows: 94°C for 5 min, 94°C for 1 min, 65°C∼55°C for 1 min, and each cycle temperature was reduced by 0.5°C for 20 cycles, 72°C for 1 min, then 94°C for 1 min, 50°C for 1 min, 72°C for 1 min with 10 cycles, and 72°C for 10 min. The PCR product was first detected by 1% agarose gel electrophoresis.

The JY-TD331A denatured gradient gel electrophoresis system was used to analyze the 16S rDNA V3 region (approximately 200 bp) with denatured 25%–65% (100% = 7 mmol/L urea and 40% deionized formamide) polyacrylamide denatured adhesive (acrylamide:bis-acrylamide = 37.5:1, W/W). The 5 μL PCR product was added to 10 μL water and 10 μL 6x loading buffer and then analyzed in the JY-TD331A type denatured gel electrophoresis system for 13 h under a voltage of 80 V and a temperature of 60°C. After electrophoresis, silver staining was conducted for 15 min. Then the gel was washed with sterile water and photographed in a JD-801 gel imaging system (JEDA Technology Development Co., Ltd, Jiangsu, China). The electrophoretogram was analyzed using Quantity One software (Bio-Rad), and the diversity of the gut microbial community of *S. litura*, including Simpson, Shannon, Pielou and Brillouin indices, were calculated.

The clear DGGE bands were cut off with a disinfected blade and transferred into a 2 mL centrifuge tube. Fifty microliters of sterile deionized water were added and bands were mashed with the head of the sterilizing pipette before being left at 4°C overnight. Five microliters of the suspension were taken as a template for PCR with the primers 343F and 534R without GC-clip. The reaction program was as follows: 95°C 0.5 min; 94°C 30 s, 50°C 30 s, 72°C 1 min for 10 cycles, with a final extension at 72°C for 10 min. A purification kit (ShengGong, Shanghai) was used to purify the PCR product. The PCR product was inserted into a plasmid of PGEM-T (Promega, Madison, WI, United States), then the plasmid was transformed into *E. coli* DH5α. The products were sequenced by Biotech Boshan Co., Ltd., Shanghai, and then aligned with the NCBI GenBank database to identify the bacterial species. In order to compare the consistency between the DGGE and metagenomics sequences, the DGGE sequences were used as queries to search the data in the metagenomics using the local blast function in Bioedit 7.1.3.0. The homolog sequences were then further identified by online Blast.

### Determination of the Effects of Antibiotics and Gut Bacteria on the Nutrition Indices of *S. litura*

Initial diet with antibiotics/bacteria preparation: (1) Preparation of diet with antibiotics: A solution of 1 mg/mL ciprofloxacin, 1 mg/mL levofloxacin and 2 mg/mL metronidazole was prepared, then the cabbage leaves were soaked in this solution for 20 min; 1% Tween-20 surfactant was added to enhance the adsorption capacity of antibiotics on cabbage leaves. The antibiotics were added to the artificial diet described above to obtained a diet with final concentration of 1 mg/mL ciprofloxacin, 1 mg/mL levofloxacin, and 2 mg/mL metronidazole. (2) Preparation of diet with gut bacteria: A gut bacterium *Enterobacter* sp. (Eb: GenBank accession number: KU841477), which was isolated from the *S. litura* gut earlier by traditional culture method (Sun et al., 2017) was propagated in LB medium. The strain has previously been shown to degrade cellulose (Sun et al., 2017). The full gut microbiome (GM), a complex bacterial community was enriched from the *S. litura* gut in LB medium: 10 healthy 4th instar larvae of *S. litura* were randomly selected, and their gut contents were collected under aseptic conditions. The gut contents were dissolved in 2 mL sterile water. Then, 100 μL of this gut-content solution was incubated in liquid LB medium overnight at 30°C and with shaking at 100 rpm. The bacterial culture was centrifuged at 5000 rpm, washed with sterile water, and the bacterial was prepared to the appropriate concentrations for subsequent experiments. The concentration of gut bacteria in the artificial diet was 1 mL OD600 = 0.1/10 g artificial diet. The cabbage leaves were socked in the bacterial solution with a concentration of OD600 = 1.0 for 20 min, and 1% Tween-20 surfactant was added to enhance the cabbage leaves’ adsorption capacity. It should be noted that although taro leaves were used in the metagenomic and DGGE studies, cabbage was used here. The reason is that the *S. litura* was collected on taro leaves, which was the season of taro planting, allowing *S. litura* to be reared on taro leaves in the laboratory. However, due to the change in season it became difficult to rear taro and therefore cabbage, which is also an important crop that *S. litura* feeds on, was used for this experiment.

Bacteria administration and growth rate analysis: thirty healthy 4th instar larvae of *S. litura* were randomly selected with fresh larvae were individually weighed before the experiment. The larvae were reared on the artificial diet or cabbage leaves with antibiotics for 24 h. After 24 h, the larvae moved to another artificial diet/cabbage leaves for 24 h without antibiotics but with the single gut bacterium *Enterobacter* sp. (Eb), or the full gut microbiome (GM). The larvae reared without antibiotics or bacteria were used as controls. Individuals were kept on this diet for 48 h, with the diet renewed at the midpoint (24 h). Any remaining food was dried at 80°C and weighed. The larvae were then starved for 6 h to permit defecation. Then, the fresh weight and dry weight of the insects were measured. The nutrition indices, such as relative growth rate (RGR) and relative consumption rate (RCR), were analyzed based on the above data. The nutrition indices were calculated by RGR = G/(B × T), RCR = I/(B × T), where G = dry weight of larvae after experiment – dry weight of larvae before experiment, I = dry weight of diet before experiment – dry weight of diet after experiment, B = (dry weight of larvae after experiment + dry weight of larvae before experiment)/2, and *T* represents the number of experimental days. The data were analyzed by one-way ANOVA, followed by an LSD *post hoc* test if the homogeneity of variance test significance was >0.05; however, the data were analyzed by Tamhane’s T2 nonparametric test when the homogeneity of variance test significance was <0.05. All analyses were conducted by SPSS 19.0 (IBM, United States).

## Results

### Metagenomic Characterization of *S. litura* Gut Microbes

A total of 7,641.81 Mb and 7,334.18 Mb raw data of Metagenomic for the gut microbes of *S. litura* feeding on taro leaf (Sl-tar) and artificial diet (Sl-art), respectively, were obtained using the Illumina HiSeq2500 platform. After filtering out low-quality sequences, 7060.84 Mb and 6830.86 Mb clean data were obtained for Sl-tar and Sl-art, respectively (Supplementary Table S1). The effective data (clean data) of Sl-tar and Sl-art samples were used for SOAP *de novo* assembly, and a total length of 17.18 Mb and 106.33 Mb contigs were identified (Supplementary Table S2). A total of 210,731 ORFs were obtained according to the contigs. The average length of ORF sequences was 565.94 bp, and the average GC content was 54.61% (Supplementary Table S3).

### Diversity of *S. litura* Gut Microbes

**FIGURE 1:**
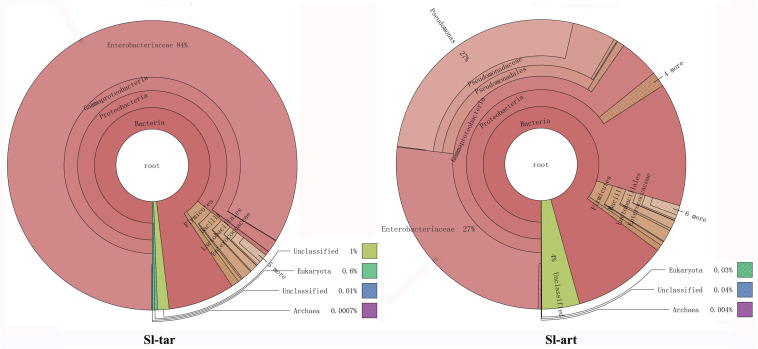
Species annotations of *S. litura* gut microbes using Krona. Circles represent different classification levels from inside to outside. Sector size represents the relative proportions of different species. Where Sl-tar are *S. litura* reared on taro leaves and Sl-art are *S. litura* reared on the artificial diet.

**FIGURE 2:**
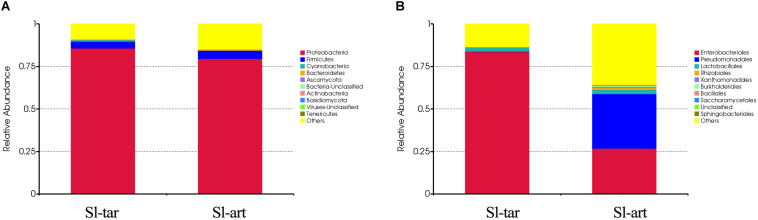
Microbe composition in the *S. litura* gut at the phylum level **(A)** and order level **(B)**. Sl-tar, *S. litura* reared on taro leaves; Sl-art, *S. litura* reared on artificial diet.

Metagenomic analysis showed that the gut bacteria of Sl-tar and Sl-art samples accounted for 98% and 96% of the total gut symbionts, respectively. The proportions of archaea, viruses and eukaryotes were relatively low (Figure 1 and Supplementary Table S4). According to the classification of the two samples of bacteria, the most abundant in the Sl-tar was Proteobacteria (85.66%), followed by Firmicutes (4.18%). The Sl-tar also included Ascomycota, Cyanobacteria, Tenericumes, Bacteroidetes, Basidiomycota, Fusobacteria, and Actinobacteria. The most abundant in the Sl-art was Proteobacteria (79.63%), followed by the Firmicutes (4.82%), it also included Actinobacteria, Bacteroidetes, Ascomycota, Tenericumes, Euryarchaeota, Cyanobacteria, and Fusobacteria (Figure 2A and Supplementary Table S4). The bacteria in the two lines (Sl-tar and Sl-art) were highly consistent with each other at the phylum level. The dominant bacteria were Proteobacteria and Firmicutes. However, at the order level, the Sl-tar was mainly composed of Enterobacteriales, which accounted for 83.67%. It also included Lactobacillales (2.37%) and Bacillales (0.11%), as well as Clostridiales and Eurotiales. The Sl-art was mainly composed of Pseudomonadales (31.95%), Enterobacteriales (26.91%) and Xanthomonadales (0.75%). It also included Lactobacillales (2.68%), Rhizobiales (0.89%), Burkholderiales (0.58%), and smaller proportions of Sphingobacteriales, Clostridiales, Actinomycetales, and Flavobacteriales. Our data indicated that the Sl-tar was mainly composed of Enterobacteriales (83.67%), while the Sl-art was mainly composed of Pseudomonadales (31.95%) and Enterobacteriales (26.91%). In addition, the proportions of other orders also differed (Figure 2B and Supplementary Table S4). These results demonstrate that although the gut bacteria of the two lines were similar at the phylum level, there was a significant difference at the order level.

Analysis based on other classification levels also showed differences between the two lines. At the class level, the Sl-tar was mainly composed of Gamma-Proteobacteria (85.16%) and Bacilli (3.61%); however, the proportion of Gamma-Proteobacteria was only 64.17% in the Sl-art (Supplementary Figure S1A and Supplementary Table S4). In the classification at the family level, the Sl-tar was mainly composed of Enterobacteriaceae (83.68%), followed by Enterococcaceae (1.53%). In addition, there were small proportions of Pseudomonadaceae, Bacillaceae, Clostridiaceae, Lachnospiraceae, Aspergillaceae, and Leptosphaeriaceae. The Sl-art was mainly composed of Pseudomonadaceae (31.49%), followed by Enterobacteriaceae (26.91%), Enterococcaceae (1.70%), and many other small proportions of Xanthomonadaceae, Brucellaceae, Comamonadaceae, Alcaligenaceae, and Sphingobacteriaceae (Supplementary Figure S1B and Supplementary Table S4). The two lines also varied greatly at the genus level (Supplementary Figure S1C and Supplementary Table S4). There was a clear difference in the composition and structure of the gut bacteria between Sl-tar and Sl-art, indicating that the bacterial diversity in the *S. litura* gut is related to its diet. Previous studies have shown that changes in the food of *Anopheles gambiae* larvae affect the structure of their gut microbes (Wang et al., 2011). However, relatively high abundances of Enterobacteriaceae, *Pseudomonas*, *Bacillus*, *Enterococcus* and *Klebsiella* were observed in the Sl-tar and the Sl-art. Venn diagram analysis showed that there were 52.7% identical orders between the two lines and 38.3% at the genus level (Supplementary Figure S2 and Supplementary Table S4), which indicate a possible core microbiota in the *S. litura* gut.

**FIGURE 3:**
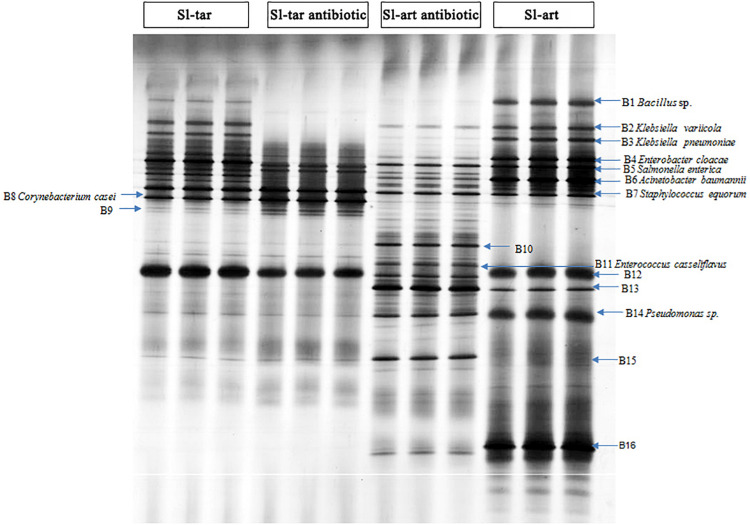
DGGE analysis of *S. litura* gut microbes. Sl-tar, *S. litura* reared on taro leaves; Sl-art, *S. litura* reared on artificial diet; Sl-tar antibiotic, *S. litura* reared on taro leaves containing antibiotics; Sl-art antibiotic, *S. litura* reared on artificial diet containing antibiotics.

Denaturing gradient gel electrophoresis (DGGE) analysis was performed on the gut bacteria of the fourth-instar larvae of Sl-tar and Sl-art (Figure 3). The results showed that there were differences in gut bacteria between the two lines of *S. litura*. The diversity index of the gut bacteria of Sl-art fed with artificial diet showed that SIMPSON (J), SHANNON (H), PIELOU and BRILLOUIN were all larger than those in Sl-tar (Supplementary Table S5), indicating the possible influence of food on the diversity of gut bacteria in *S. litura*.

The electrophoresis bands from DGGE were cut and sequenced. The results indicated that the dominant bacteria were Proteobacteria, Firmicutes, and Actinobacteria at the phylum level, which contained the families of Enterobacteriaceae, Corynebacteriaceae, Bacillaceae, Staphylococcaceae, and Pseudomonadaceae (Supplementary Table S6). The abundance of *Bacillus* (band 1) and *Pseudomonas* (band 14), especially *Pseudomonas*, were significantly higher in the Sl-art than in the Sl-tar (Figure 3). This result is consistent with the above metagenomic sequencing results, again suggesting that food affects the diversity of gut bacteria in *S. litura*. The DGGE sequences were used as queries to extract homolog 16S rRNA from metagenomic (SI-tar and SI-art) data using the local blast. Only 5 and 10 homolog sequences were extracted from SI-tar and SI-art metagenomic, respectively. However, only 1 sequence in SI-tar metagenomic was matched to *Klebsiella pneumoniae*, with other sequences matching different species identified by blast in NCBI. Similarly, there were only two sequences matched to *Pseudomonas* sp. and one sequence matched to *Acinetobacter* sp. in SI-art metagenomic, but other sequences were different with the DGGE.

### Functional Analysis of *S. litura* Gut Microbes

**FIGURE 4:**
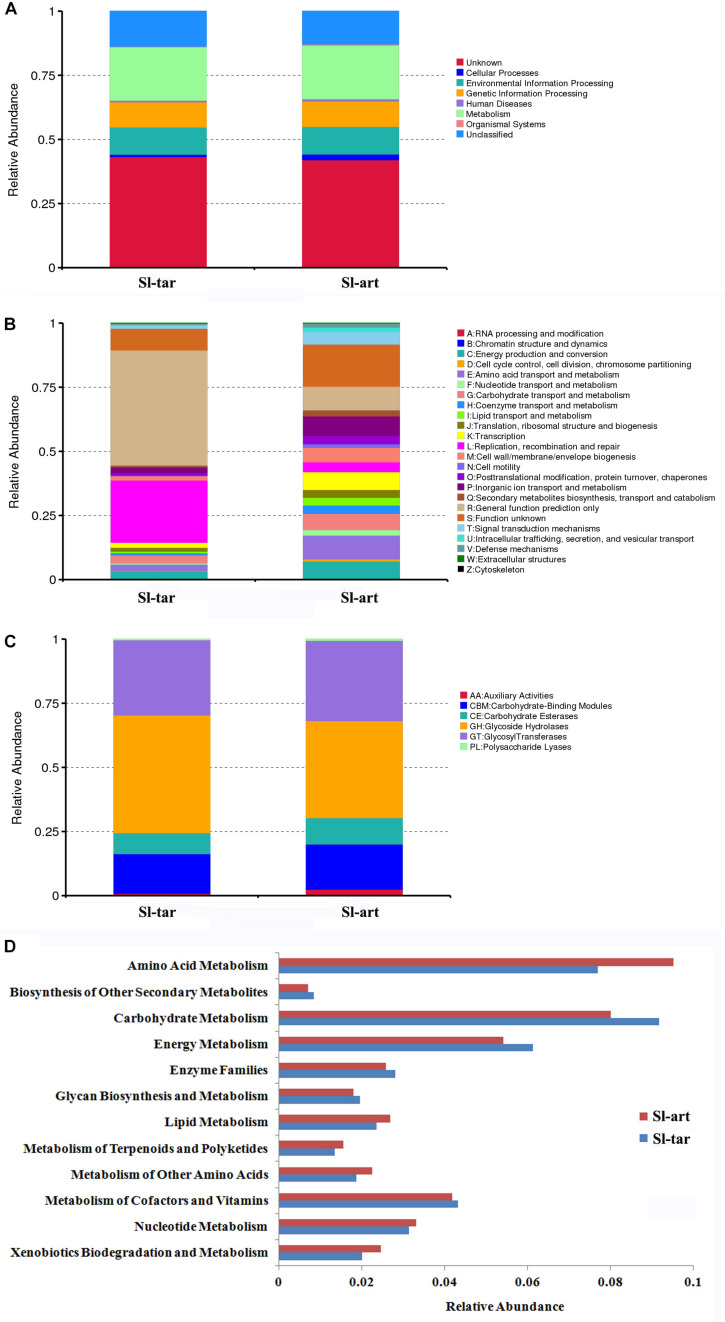
Metagenomic analysis of gut microbes in *S. litura*. **(A)** KEGG analysis of *S. litura* gut microbes, **(B)** eggNOG analysis of *S. litura* gut microbes, **(C)** CAZy analysis of *S. litura* gut microbes, **(D)** KEGG metabolic functions of the *S. litura* gut microbes.

Metagenomics of the *S. litura* gut bacteria were analyzed. Using MetaGene, 210,731 open reading frames (ORFs) were obtained (Supplementary Table S3). The ORFs were analyzed using the KEGG^1^, eggNOG (Huerta-Cepas et al., 2016) and CAZy databases (Lombard et al., 2014). The results showed that the gene abundance in the first KEGG level (Cellular Processes, Environmental Information Processing, Genetic Information Processing, Human Diseases, Metabolism, Organismal Systems) was not obviously different between Sl-tar and Sl-art (Figure 4A). However, the functional clustering of the two samples was quite different in eggNOG classification, the “General function prediction only,” “Replication, recombination and repair” showed the greatest difference, and both of these two functions were more abundant in Sl-tar than Sl-art. On the contrary, the metabolic functions of “Amino acid transport and metabolism” and “Secondary metabolites biosynthesis, transport and catabolism” were all higher in Sl-art (Figure 4B). CAZy analysis found that the GHs gene abundance in the Sl-tar line was higher than that in the Sl-art line (Figure 4C). Further analysis of the metabolic function of KEGG revealed that the genes involved in metabolism accounted for the largest proportion (approximately 21%) of the two samples, with the highest abundance of genes involved in carbohydrate and amino acid metabolism. The Sl-tar had more abundant carbohydrate metabolism genes, but less amino acid metabolism than the Sl-art (Figure 4D). The results were in accordance with the GH gene abundance in the Sl-tar, which was higher than that of Sl-art as analyzed by CAZy (Figure 4C).

### Biodegradation of Carbohydrates by Gut Bacteria of *S. litura*

In this study, the contigs were used to identify gene information for functional annotation by MetaGeneMark software, then a series of enzyme-encoding genes involved in the degradation of cellulose, xylan and pectin were identified by KEGG analysis (Supplementary Table S7). According to the LCA algorithm, *Enterococcus* and *Pseudomonas* encoded endoglucanases that participate in the degradation of cellulose. *Enterococcus* encodes xylan 1,4-beta-xylosidase, which degrades xylan. A pectinesterase and a polygalacturonase, which are involved in the degradation of pectin, were encoded by *Enterobacteriaceae* and *Enterococcus*. The *Enterococcus casseliflavus* and *Enterococcus mundtii* may play important roles in the degradation of these polymers. Enterobacteriaceae and *Pseudomonas*, which were more abundant in the gut of *S. litura*, were also involved in this process (Supplementary Table S7). The enzymes that participate in cellulose, xylan and pectin biodegradation all existed in both the Sl-tar and the Sl-art.

CAZy database analysis showed that the gut microbes of *S. litura* were rich in carbohydrate degradation-related genes, including 93 families of GHs, 55 families of glycosyltransferases (GTs), 15 families of polysaccharide lyases (PLs), 12 families of carbohydrate esterases (CEs), 9 families of auxiliary activities (AAs), and 38 families of carbohydrate binding modules (CBMs) (Supplementary Table S8). GHs play an important role in cellulose degradation, and it was much higher in *S. litura* than *P. xylostella* (55 families of GHs) (Xia et al., 2017). Compared with the GH family of rumen microbes of *B. grunniens* (Dai et al., 2012) and the gut microbes of *N. exitiosus* (Warnecke et al., 2007), *A. mellifera* (Engel et al., 2012) and *P. xylostella* (Xia et al., 2017), the diversity of gut GH genotypes and gene abundance increased with increasing complexity of the dietary plant fibers. Bees feed on pollen, a food that is relatively simple and easy to degrade compared with other food stuffs; thus, the GH family has the lowest diversity (Supplementary Table S9). Another interesting point is that although the total abundance of GHs in the Sl-tar was higher than that in the Sl-art, the gene in Sl-art was much diverse (Supplementary Table S9), indicating that there may be different mechanisms of carbohydrate digestion in these two lines.

### Metabolic Degradation of Plant Secondary Metabolites by Gut Bacteria of *S. litura*

In the gut microbes metagenomic of *S. litura*, the KEGG pathway of “Benzoate degradation” which belongs to “Xenobiotics biodegradation and metabolism” was enriched. Catechol is an important central molecule in the “Benzoate degradation pathway.” The simple structure of catechol may become the core of many complex secondary metabolites; therefore, the ability to degrade catechol indicates the potential ability of gut microbes to degrade some other plant secondary metabolites (Ellis, 1971; Arunachalam et al., 2003; Qualley et al., 2012; Xia et al., 2017). A complete catechol metabolic pathway was present in the gut microbial metagenomic of *S. litura* (Supplementary Table S10). These enzyme genes were mainly encoded by *Pseudomonas*, *Acinetobacter*, Enterobacteriaceae, and *Enterococcus* (Supplementary Table S10). Among them, *Pseudomonas* may play an important role in this pathway, as it encodes 9 out of 15 KO genes in the Catechol biodegradation pathway (Supplementary Table S10). Interestingly, although the numbers of degradation genes in the Sl-tar line was fewer than that in Sl-art, the total abundance of the Sl-tar was close to that of Sl-art (Supplementary Table S10), indicating that the two lines enrich genes in different ways. Thus, the degradation mechanism of plant secondary metabolites may be different between Sl-tar and Sl-art, although the specific mechanism needs to be further verified by functional experiments in the future.

### Role of Gut Bacteria in Amino Acid Synthesis of *S. litura*

Based on the metagenomic analysis, we found that the gut bacteria may cooperate to synthesize essential amino acids such as histidine, threonine, valine, leucine, isoleucine, phenylalanine and tryptophan (Supplementary Figure S3). They also participated in the synthesis of non-essential amino acids such as alanine, tyrosine, serine and glycine (Supplementary Figure S3). The enzymes involved in amino acid synthesis were mainly encoded by Enterobacteriaceae, *Pseudomonas*, *Enterococcus casseliflavus*, *Acinetobacter*, etc. (Supplementary Table S11). The *Pseudomonas* genus showed great potential for amino acid synthesis as it possesses half of the enzymes of histidine biosynthesis, and three-quarters of the enzymes for valine/leucine/isoleucine biosynthesis. The Enterobacteriaceae encode all the genes for valine/leucine/isoleucine biosynthesis and various genes associated with other amino acids (Supplementary Table S11). The bacteria that participate in the amino acid biosynthesis were the most abundant in the gut of *S. litura*, suggesting that the gut bacteria of *S. litura* may play a role in the supply of essential amino acids.

### Effect of Antibiotics and Gut Bacteria on Feeding and Growth of *S. litura*

After feeding on an antibiotic-supplemented diet, DGGE analysis showed a reduced abundance of gut bacteria in both the Sl-tar and Sl-art (e.g., bands of B1, B2, B3, B4, B6, B12, B14, B16). However, the reduction was more pronounced in the Sl-art group. This could be attributed to the greater stability and uniformity of antibiotic administration possible through an artificial diet as compared to leaf material (Figure 3). It is interesting to note that some species of bacteria (bands of B10, B11, A13, B15) with low abundance in antibiotic-free food in the Sl-art were significantly increased after antibiotic treatment (Figure 3). Sequence verification showed that B11 was *E. casseliflavus*, but none of the other three species had been effectively amplified and sequenced. The enrichment of these bacteria may be due to their strong resistance to antibiotics, and whether the enrichment of these bacteria affects the function of their host remains unclear.

**FIGURE 5:**
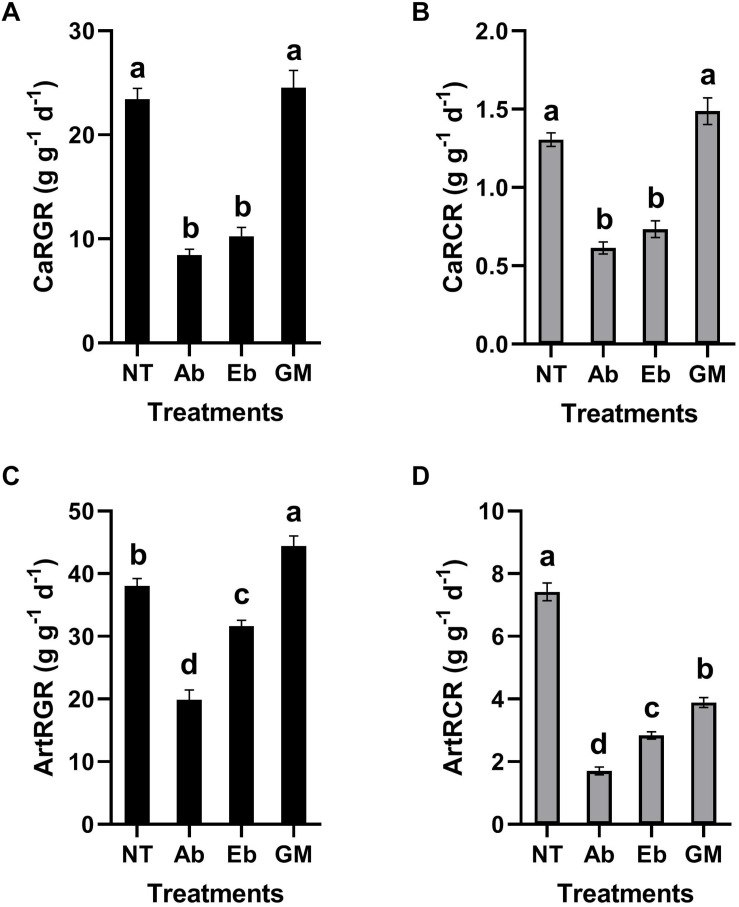
Effect of gut bacteria and antibiotics on nutrition indicators of *S*. *litura*. **(A)** Relative growth rate (RGR) of *S. litura* reared on cabbage, **(B)** Relative consumption rate (RCR) of *S. litura* reared on cabbage, **(C)** Relative growth rate (RGR) of *S. litura* reared on the artificial diet, **(D)** Relative consumption rate (RCR) of *S. litura* reared on the artificial diet. NT: *S. litura* reared with no treatment; Ab: *S. litura* reared with antibiotics; Eb: *S. litura* reared on diet containing the bacteria of *Enterobacter* sp. (GenBank accession number: KU841477) which was previously isolated from the *S. litura* gut; GM: *S. litura* reared on diet that contained bacteria of the full gut bacterial community (a complex bacterial community enriched from the *S. litura* gut in LB medium).

Antibiotic treatment had significant effects on the nutritional indices of *S. litura* larvae. Compared with the control group, the RGR and RCR of the larvae were all decreased significantly no matter whether the insect was reared on the artificial diet or leaf material with antibiotics (Ab) (Figure 5). Interestingly, the RGR and RCR increased when bacteria were reintroduced to *S. litura*, to some extent alleviating the fitness cost associated with the antibiotic treatment. However, the recovery ability was strongest when we reintroduced the full gut microbiota (GM: a complex bacteria community enriched from *S. litura* gut by LB medium) than only single strain of *Enterobacter* sp. (Eb: GenBank accession number: KU841477) (Figure 5).

## Discussion

The diversity of gut bacteria in the 4th instar larvae of *S. litura* was analyzed by metagenomic sequencing and DGGE. The results of both methods showed that Proteobacteria and Firmicutes were dominant phyla in the gut of *S. litura*, and the abundance of Proteobacteria was higher than that of Firmicutes. It was found that the DGGE sequences did not match the metagenomic sequences well. The possible reason is that these different approaches are measuring different aspects of the microbiota. Metagenomics may not be able to detect all 16S rRNA sequences due to the depth of sequencing and assembly. Even if some bacteria 16S rRNA have not been detected, other genes could still annotate corresponding species. Due to the short sequences of DGGE, the difference between the annotation results of the two methods is normal. Even though DGGE samples were collected side-by-side from the same batch of insects, and may not be considered as biological replicates, the samples used for metagenomic analysis were from different batches. It is difficult to achieve the absolute unity between the different samples analyzed by two methods, but they complement and verify each other. Another noteworthy thing is that 75% alcohol was used to disinfect the body surface in this study. Due to the complexity of the body surface of insects, it cannot be guaranteed that all the microbes on the body surface were completely removed. Therefore, it is suggested that 1% chlorine can be further added to disinfect the body surface in the future study to eliminate the impact of microbial pollution as much as possible. In addition, contigs were used for species annotation and gene abundance calculation in this study. In the future, ORFs based analysis may be better to avoid the recovery of false positives in different samples.

The gut bacteria of *S. litura* feeding on taro leaves differed greatly from those feeding on the artificial diet at the family level. Enterobacteriaceae dominated the gut bacteria of *S. litura* feeding on taro leaves, while Pseudomonadaceae dominated the gut bacteria of *S. litura* feeding on the artificial diet. Similar results have been found with silkworm in which differences in the gut microbes are seen for those feeding on mulberry leaves or artificial diet with these differences leading changes in nutritional metabolism and immune resistance (Dong et al., 2018). A study in pine processionary moth, *Thaumetopoea pityocampa*, also revealed that different host plants lead to a different gut bacterial community (Strano et al., 2018). These finding in Lepidoptera support evidence found across a range of insect orders demonstrating the importance of diet on gut microbial diversity (Chandler et al., 2011; Perezcobas et al., 2015; Paniagua Voirol et al., 2018). Additionally, feeding habits, such as the extent of polyphagy can also greatly change the diversity of gut microbiota. Initial comparison with the oligophagous *P. xylostella* shows the larvae gut was also dominated by Proteobacteria and Firmicutes with Proteobacteria more abundant than Firmicutes (Xia et al., 2013, 2017). However, differences are seen when the bacteria are compared at the family level. *Spodoptera litura* were dominated by Enterobacteriaceae when reared on taro leaves but Pseudomonadaceae and Enterobacteriaceae on the artificial diet while *P. xylostella* larvae were dominated by Enterobacteriaceae, which was mainly composed of *Enterobacter cloacae* and *Enterobacter asburiae* (Xia et al., 2017). It seems that *S. litura* and *P. xylostella* possess different core microbiota. Moreover, the composition of gut microbes of *S. litura* (24 phylum, 801 bacteria species in the metagenomic, Supplementary Table S4) is more complicated than that seen in *P. xylostella* (10 phylum, 137 bacteria species in the metagenomic) (Xia et al., 2017), which may result from the difference of food complexity. In the same way, honeybees feed on nectar, a structurally simpler food source, resulting in a simpler gut microbial structure (Engel et al., 2012). On the contrary, the polyphagous tobacco budworm, *Heliothis virescens*, has core gut microbes resembling *S. litura* due to its composition of Enterobacteriaceae, Enterococcaceae and Pseudomonadaceae (Staudacher et al., 2016). Yun et al. (2014) found that the gut bacteria diversity in omnivorous insects was higher than that in stenophagous (carnivorous and herbivorous) insects by comparing 218 insect species.

Paniagua Voirol et al. (2018) compared 30 lepidopteran insect species through which they found that Enterobacteriaceae, Pseudomonadaceae and Bacillaceae, as well as the corresponding genus of *Enterobacter*, *Pseudomonas*, and *Enterococcus* are the core flora in the gut of Lepidoptera. However, due to the influence of diet, gut physiology, environment and developmental stage, it is common to find significant variability across different species and even within a species. The core gut microbiota of *S. litura* observed in this study are in line with what has been observed in other Lepidoptera species. Despite this, the differences between species are still significant, even among very closely related species. For example, in *S. littoralis* the gut microbiota is dominated by Proteobacteria and Firmicutes; however, the Proteobacteria dominated at the egg and adult stages, whereas Firmicutes dominated at the late-instar larval and pupal stages. At the genus level, *Pantoea* dominated the egg and female adult but *Enterococcus* dominated at the larval and pupal stages. These results are notably different from our findings in *S. litura*, despite the species being closely related. However, diet alone is not responsible for dictating gut microbiota as *Acronicta major*, *Diaphania pyloalis* and *B. mori* that all feed on mulberry are found to have species-specific compositions, which is likely to be correlated with host biology (Chen et al., 2018). Therefore, we speculate that the difference between *S. litura* and *S. littoralis* may not only be caused by the difference in diet but species specificity is likely to play a role. The species-specific core microbiota in these insects may linked to their physiology or other important biological characteristics.

By analyzing the gut bacterial metagenomics of *S. litura*, we found that gut bacteria may be involved in various metabolic pathways, such as the detoxification of plant secondary metabolites, amino acid synthesis, and cellulose and carbohydrate degradation. Plants can produce numerous secondary metabolites, such as phenols and terpenoids, to resist phytophagous insects and pathogenic microbes (Ellis, 1971; Bennett and Wallsgrove, 1994). To counter this, phytophagous insects must develop a defense system equip to deal with these plant-produced secondary metabolites (Simon et al., 2015). Previous studies have found that gut microbes play an important role in this process (Ceja-Navarro et al., 2015). The complete catechol metabolic pathway present in the gut microbial metagenomics of *S. litura* indicated the potential function in the detoxification of plant secondary metabolites. As the possibility of cooperation of amino acid biosynthesis, it should be noted that based on current data we do not know if a specific pathway is complete or partially present on the different family/genus/species listed in the Supplementary Table S11. Because of the sequencing depth and sequences comparison of the metagenomics, there are many genes that cannot be assigned to specific genera or species. In addition, it cannot be ruled out that some genes are not detected in the metagenomic sequencing. Thus, from the current results, it is difficult to assess if the bacteria cooperate (shared steps from the same pathway) to produce amino acids. It is possible materials are released into the environment and utilized by other bacteria or production occurs solely for their own requirements. Data generated in this study identifies the possible functions of these microbes but deeper sequencing and further functional verification is required to confirm the specific mechanisms involved.

A series of enzymes involved in food digestion such as cellulose-degrading enzymes, GHs, and polysaccharide lyases were found in the databases of CAZy and KEGG. Carbohydrates are some of the most important organic compounds in nature as an energy source for the life-sustaining activities of organisms. To obtain the necessary nutrients, phytophagous insects need to breakdown the plant cell walls (Prins and Kreulen, 1991; Calderón-Cortés et al., 2012). Plant cell walls are rich in cellulose, xylan, and pectin which are difficult to degrade (Whitney et al., 1999). The role of termite gut microbes in helping the host to digest plant lignocellulose and thus promoting host-plant coevolution is the most typical example in this field (Varma et al., 1994; Hongoh, 2011; Yuki et al., 2015). The endosymbiotic bacteria of pollinators contain pectin-degrading genes allowing them to obtain energy from the source (Engel et al., 2012). Another study in the cricket, *Acheta domesticus*, showed that the gut bacteria increased the digestive efficiency of plant polysaccharides (Kaufman and Klug, 1991). It is suggested a lack of cellulose degrading enzyme genes in Lepidoptera means they are reliant on these endosymbionts for effective breakdown of plant materials (Paniagua Voirol et al., 2018). The strains of Enterobacteriaceae, *Pseudomonas* and *Enterococcus* (*E. casseliflavus* and *E. mundtii*) in the *S. litura* gut were mainly involved in the coding of these enzymes and the metabolism of carbohydrates. The large number of glycan degrading enzyme genes in gut microbes of *S. litura* suggest that they play an important role in the host digestion and growth. The Sl-tar had more carbohydrate metabolism genes than the Sl-art. It is possible that the Sl-tar requires more genes related to carbohydrate metabolism to degrade and adapt to plants than the Sl-art fed on artificial diet which may be composed fewer carbohydrates, such as cellulose.

Glycoside hydrolases (GH) are an important enzyme family in polysaccharide degradation. A comparative study of GH in the gut of herbivores with different food complexity suggested that the diversity of gut GH genotypes was highly correlated with the complexity of dietary plant fibers. Oligophagous insects such as *P. xylostella*, which mainly feeds on cruciferous plants, has a much simpler GH composition than that of *S. litura*, suggesting that polyphagous insects may need more carbohydrate hydrolase to help the host digest and degrade food. The GH diversity of *S. litura* was similar to that of *B. grunniens*, although the latter had more abundant GH genes (Supplementary Table S9) which may indicate greater proficiency in food digestion. It should be emphasized that the composition of the GH family in the termite gut is simple, even less diverse than that of *P. xylostella*. It is possible that this is because metagenomics data for termites was achieved with first-generation sequencing and therefore coverage may have been relatively low compared to species analyzed using second-generation sequencing. In this comparison, we did not control that all samples were in the same sequencing depth. For these reasons we advise caution in comparing the results across different experimental methods and interpretation should contain this caveat.

The determination of nutrient indices of phytophagous insects can directly reflect their feeding and growth. This is a common method to study the relationship between phytophagous insects and food (Scriber and Slansky, 1981). Based on the metabolic potential of gut microbes, and the high abundance of Enterobacteriaceae in the gut of *S. litura*, the study focused on the strain of Enterobacter due to its relationship with digestion and growth in *S. litura*. At the same time, the whole gut microbes of *S. litura* cultured *in vitro* was also used for comparative study as it is recognized that the gut bacteria often function as a community. Our study showed that antibiotic treatment affected the diversity of gut bacteria of *S. litura* and the nutrient indices. The results showed that the RGR and RCR of *S. litura* were significantly reduced after antibiotic treatment. Whether antibiotics inhibit the feeding and growth of *S. litura* due to the direct effect or the indirect effect of the elimination of gut bacteria is yet unknown. However, the recovery of gut bacteria improved the nutritional index. Most notably the recovery of full gut bacteria had a greater impact than that of a single bacteria strain, indicating that the microbiota as a whole play an important role in the host’s feeding and growth. The exact mechanisms of how gut bacteria affect food intake, digestion, absorption, and metabolism of *S. litura* are still to be elucidated. Further considerations may be the association that gut bacteria may be having to geographical distribution of the pest and potential side-effects of antibiotic administration on *S. litura*.

It is noteworthy that although RGR and RCR were consistent in a cabbage diet (Figures 5A,B), they were inconsistent in the artificial diet (Figures 5C,D). In the artificial diet, the GM treatment restored or even improved the RGR, however, it was not reflected on RCR as the GM in RCR was significantly lower than the control. A potential explanation is that in artificial diet the gut microbes contribute to more efficient digestion and metabolism.

Studies in *Drosophila melanogaster* have found that gut microbes can promote the nutrition and metabolism of the host, thus enhancing the development rate of larvae (Ridley et al., 2012). Another study in the butterfly, *Melitaea cinxia*, hypothesizes that the main difference in growth rate of the butterfly on different plants is due to changes in the gut microbiota, or its interaction with the host plant species (Ruokolainen et al., 2016). Our work builds on what has been shown previously; that the gut microbiota perform vital metabolic functions and are closely linked to the host’s growth and development.

There is work which contradicts these findings. A study by Thakur et al. (2016) showed that adding antibiotic, streptomycin sulfate, to the artificial diet of *S. litura* changed the gut microbial diversity, decreased mortality, and shortened development duration. In addition, they found that the antibiotics significantly promoted the nutrient indices RGR and RCR. They also isolated *E. cloacae* bacteria from the gut, however, reintroducing this bacterium to *S. litura* by feeding on treated castor leaves significantly decreased the nutrient indices and even caused significant mortality (Thakur et al., 2015). We speculate that there may be several reasons that the work by Thakur et al. (2015) yielded such different results: (1) Different foods were used in the experiments. The microbes attach to the food and the characteristics of the food may directly affect host physiology. (2) Different antibiotics and concentrations have different physiological effects on host insects. The antibiotics in the artificial diet can remove pathogenic bacteria and protect the host, which may be the reason why antibiotics promoted host feeding in the work by Thakur et al. (2015). (3) Antibiotics may have removed mutualistic/commensal species, promoting invasion by pathogenic bacteria now facing reduced competition. This could explain the decrease in fitness observed in our study. (4) Different bacteria have different functions, e.g., *E. cloacae* was a pathogen in Thakur et al.’s (2015) study, but we found that *E. cloacae* to have an important function in *P. xylostella* (Xia et al., 2017). Engel et al. (2012) suggest that a difference in 16S rRNA smaller than 1% between subspecies, may lead to different functions for the host.

In summary, the high-throughput DNA sequencing allowed us to systematically and comprehensively analyze diversity of the gut microbiota of *S. litura*. The potential functions of gut bacteria in *S. litura* contributing to food digestion, nutrition, and metabolic detoxification were analyzed and the functions of gut bacteria in nutrition physiology were preliminarily verified. However, the exact mechanisms of gut bacteria participation in these roles remains unclear. While we have established candidate microbes and roles, these require further study for verification. Our work provides a perspective and direction for further study on the functions of the gut microbiota of the important pest species *S. litura*.

## Data Availability Statement

The datasets generated for this study can be found in the raw sequences of the *S. litura* gut microbiota were deposited in the SRA (Sequence Read Archive) database under accession number SRP116674.

## Author Contributions

XX and MY designed the project and analyzed the data. XX, BL, XT, and JL conducted the experiment and analyzed the data. XX and MY wrote and edited the manuscript. All authors contributed to the article and approved the submitted version.

## Conflict of Interest

The authors declare that the research was conducted in the absence of any commercial or financial relationships that could be construed as a potential conflict of interest.

## Footnotes

1 https://www.kegg.jp/kegg/
