# Supplementary material for: Characterization of Spodoptera litura Gut Bacteria and Their Role in Feeding and Growth of the Host
Source: Front Microbiol. 2020 Jun 30;11:1492. doi: 10.3389/fmicb.2020.01492 (PMC7344319; doi:10.3389/fmicb.2020.01492)
Supplement: Supplementary file 1 [file Data_Sheet_1.zip › Data Sheet 1.doc]

**Supplementary Materials**

**Characterization of *Spodoptera litura* gut bacteria and their role in feeding and growth of the host**

Xiaofeng Xia^1,2,3#^，Bomiao Lan^1,2,3,4#^，Xinping Tao^1,2,3^，Junhan Lin^1,2,3,5^， Minsheng You^1,2,3*^

^1^State Key Laboratory of Ecological Pest Control for Fujian and Taiwan Crops, Institute of Applied Ecology, Fujian Agriculture and Forestry University, Fuzhou 350002, China

^2^Joint International Research Laboratory of Ecological Pest Control, Ministry of Education, Fuzhou 350002, China

^3^Key Laboratory of Integrated Pest Management for Fujian-Taiwan Crops, Ministry of Agriculture, Fuzhou 350002, China

^4^Quanzhou Institute of Agricultural Sciences, Quanzhou 362212, China

^5^Fujian Vocational College of Bioengineering, Fuzhou 350002, China

* Correspondence: msyou@fafu.edu.cn

\# These authors contributed equally to this work.

**This file includes:**

Supplementary Figures 1-3

Supplementary Tables 1-3, 5-7, 9-11

**Supplementary Figure 1. Proportional composition of microbiota in the *S. litura* gut at the class level (A), family level (B) and genus level (C)**. Sl-tar = *S. litura* reared on taro leaves; Sl-art = *S. litura* reared on artificial diet.

**Supplementary Figure 2. Venn diagram analysis of the gut bacteria between Sl-tar and Sl-art.** A: order level; B: genus level; Sl-tar = *S. litura* reared on taro leaves; Sl-art = *S. litura* reared on artificial diet.

**Supplementary Figure 3. Proposed schematic of the biosynthetic pathways of amino acids by *S. litura* gut bacteria.** Red: essential amino acids; pink: non-essential amino acids; green: enzymes involved in amino acid biosynthesis.

**Supplementary Table 1** Metagenomic sequencing data of the gut microbiota of *S. litura*

| Sample | InsertSize（bp） | RawData（Mb） | CleanData（Mb） | Clean_Q20 | Clean_Q30 | Clean_GC（%） | Effective（%） |
| --- | --- | --- | --- | --- | --- | --- | --- |
| Sl-tar | 300 | 7,641.81 | 7060.84 | 94.39 | 89.32 | 49.39 | 92.397 |
| Sl-art | 300 | 7,334.18 | 6830.86 | 94.04 | 88.46 | 57.9 | 93.137 |

Note：InsertSize（bp）represents the use of a 300 bp Library; RawData: Raw Sequencing Data; CleanData: the valid data after filtered；Clean_Q20, Clean_Q30 represents the percentage of base numbers in CleanData with a sequence error rate less than 0.01 (mass value greater than 20) and 0.001 (mass value greater than 30); Clean_GC(%) denotes the GC content of bases in CleanData; Effective (%) represents the percentage of CleanData to RawData.

***Supplementary Table*** ***2*** *SOAP denovo assembly of the metagenomics of S. litura gut microbiota*

| SampleID | Total len.（Mb） | Num. | Average len.（bp） | N50 Len.（bp） | N90 Len.（bp） | Max len.（bp） |
| --- | --- | --- | --- | --- | --- | --- |
| The contigs information after filtering by length 500 bp: | | | | | | |
| Sl-tar | 17.18 | 12,458 | 1,378.68 | 2,106 | 557 | 174,828 |
| Sl-art | 106.33 | 57,589 | 1,846.30 | 2,906 | 699 | 385,485 |
| The contigs information after filtering by length 300 bp: | | | | | | |
| Sl-tar | 34.37 | 58,409 | 588.4 | 499 | 332 | 174,828 |
| Sl-art | 122.42 | 98,892 | 1,237.93 | 2,159 | 444 | 385,485 |

Note：Total Len. (bp): the total length of Contigs assembled; Num.: the total number of Contigs assembled; Average Len. (bp): the average length of Contigs; N50, N90 denotes that Contigs are sorted by length, and then added from length to short, when the sum value reaches 50% or 90% of the total length of Contigs; Max Len.: the length of the longest Contigs assembled.

**Supplementary Table 3** Gene prediction of the gut microbiota metagenomics in *S. litura*

| ORFs NO. | Total Len.（Mbp） | Average Len.（bp） | GC percent |
| --- | --- | --- | --- |
| 210, 731 | 119.26 | 565.94 | 54.61 |

ORFs NO.: the number of predicted ORFs; Total Len. (Mbp): the total length of predicted ORFs; Average Len.: the average length of ORFs; GC Percent: the GC content of predicted ORFs.

**Supplementary Table 5** The average of bacterial diversity indices calculated from the DGGE bands using Quantity One software

| Samples | Simpson | Shannon |
| --- | --- | --- |
| Sl-tar | 0.862 | 3.109 |
| Sl-tar antibiotic | 0.794 | 2.412 |
| Sl-art | 0.879 | 3.184 |
| Sl-art antibiotic | 0.914 | 3.568 |

Sl-tar = *S. litura* reared on taro leaves; Sl-art = *S. litura* reared on artificial diet; Sl-tar antibiotic = *S. litura* reared on taro leaves containing antibiotics; Sl-art antibiotic = *S. litura* reared on artificial diet containing antibiotics.

**Supplementary Table 6** Identification of 16S rDNA recovered from the bands in

DGGE bacteria fingerprints

| Isolated strains | GenBank Accession No. | Closest related species  (Accession No.) | Identity/% |
| --- | --- | --- | --- |
| Band 1 | KU841466 | *[Bacillus](http://blast.ncbi.nlm.nih.gov/Blast.cgi#alnHdr_545121551)* sp. (MK881067) | 100 |
| Band 2 | KU841467 | *Klebsiella variicola* (MK894865) | 100 |
| Band 3 | KU841469 | *[Klebsiella pneumoniae](http://blast.ncbi.nlm.nih.gov/Blast.cgi#alnHdr_378976159)* (MK894864) | 100 |
| Band 4 | KU841468 | *[Enterobacter cloacae](http://blast.ncbi.nlm.nih.gov/Blast.cgi#alnHdr_296100371)* (LT838172) | 100 |
| Band 5 | KU841470 | *[Salmonella enterica](http://blast.ncbi.nlm.nih.gov/Blast.cgi#alnHdr_16758993)* (MK886517) | 100 |
| Band 6 | KU841471 | *[Acinetobacter baumannii](http://blast.ncbi.nlm.nih.gov/Blast.cgi#alnHdr_491117977)* (CP040087) | 100 |
| Band 7 | KU841472 | *[Staphylococcus equorum](http://blast.ncbi.nlm.nih.gov/Blast.cgi#alnHdr_392972635)* (MK796070) | 100 |
| Band 8 | KU841475 | *[Corynebacterium casei](http://blast.ncbi.nlm.nih.gov/Blast.cgi#alnHdr_754107608)* (MK253328) | 100 |
| Band 11 | KU841473 | *[Enterococcus casseliflavus](http://blast.ncbi.nlm.nih.gov/Blast.cgi#alnHdr_558684762)* (MK517636) | 100 |
| Band 14 | KU841474 | *[Pseudomonas](http://blast.ncbi.nlm.nih.gov/Blast.cgi#alnHdr_66043271)* sp. (MH018902) | 100 |

Isolated strains: the bands isolated from the DGGE fingerprints, Closest related species: the species in NCBI GenBank which were most closest to the isolated DGGE strains by NCBI Blast analysis.

**Supplementary Table 7** Cellulose, xylan and pectin biodegradation genes in *S. litura* gut bacteria

| KO ID | Pathway | Known activities | Abandance | | Midgut community | | Genus |
| --- | --- | --- | --- | --- | --- | --- | --- |
|  |  |  | Sl-tar | Sl-art | Sl-tar | Sl-art |  |
| K01179 | Cellulose biodegradation | endoglucanase | 2.75E-05 | 0.000389 | 9 | 21 | *Enterococcus mundtii*  *Xanthomonas campestris*, *Bacillus*,  *Pseudomonas*, Enterobacteriaceae |
| K01188 | Cellulose biodegradation | beta-glucosidase | 0.00021 | 0.000307 | 18 | 40 | *Enterococcus casseliflavus*, Xanthomonadaceae, *Gilliamella apicola* |
| K01198 | Xylan biodegradation | xylan 1,4-beta-xylosidase | 0.000305 | 0.00013 | 7 | 20 | *Enterococcus casseliflavus*, *Enterococcus mundtii*, Enterobacteriaceae,  *Stenotrophomonas*, *Xanthomonas*, *Bacillus* |
| K01051 | Pectin biodegradation | pectinesterase | 0.000304 | 0.000168 | 8 | 18 | Xanthomonadaceae, Enterobacteriaceae,  *Enterococcus casseliflavus*, |
| K01184 | Pectin biodegradation | polygalacturonase | 6.61E-06 | 7.42E-06 | 1 | 1 | *Enterobacteriaceae* |
| K01213 | Pectin biodegradation | galacturan 1,4-alpha-galacturonidase | 1.97E-05 | 1.73E-07 | 1 | 1 | *Bacteria* |

KO ID: KEGG ID; Pathway: the KEGG pathway of the orthology participated in; Known activities: the activities of the enzymes; Abundance: Abundance of the gene in the gut bacteria Metagenomic; Midgut community: the number of genes detected in the Metagenomic of *S. litura* gut bacteria; Genus: the *S. litura* gut bacteria which encode the genes.

**Supplementary Table 9** Profiles of GH genes in the *S. litura* gut bacteria compared to other species analyzed by CAZy database

| CAZy Family | Abundance | | Community | | | | | |
| --- | --- | --- | --- | --- | --- | --- | --- | --- |
|  | Sl-tar | Sl-art | Sl-tar | Sl-art | *B. g*^[1]^ | *N. e*^[2]^ | A. m^[3]^ | *P. x*^[4]^ |
| Cellulases | | | | | | | | |
| GH5 | 0.00349 | 0.009581 | 28 | 72 | 1302 | 56 | 3 | 34 |
| GH6 | 0 | 0 | 0 | 0 | 0 | 0 | 0 | 0 |
| GH7 | 0 | 0 | 0 | 0 | 0 | 0 | 0 | 0 |
| GH8 | 2.72E-05 | 0.0037 | 15 | 45 | 174 | 5 | 0 | 15 |
| GH9 | 0.000307 | 0.000741 | 10 | 14 | 767 | 9 | 0 | 1 |
| GH44 | 0 | 0 | 0 | 0 | 0 | 6 | 0 | 0 |
| GH45 | 0 | 0.000551 | 0 | 2 | 13 | 4 | 0 | 0 |
| GH48 | 0 | 0 | 0 | 0 | 32 | 0 | 0 | 0 |
| Total | 0.0038242 | 0.014573 | 53 | 133 | 2288 | 80 | 3 | 50 |
| Endohemicellulases | | | | | | | | |
| GH10 | 0.000272 | 0.001794 | 9 | 16 | 2664 | 46 | 0 | 0 |
| GH11 | 0 | 0.000152 | 0 | 2 | 244 | 14 | 0 | 0 |
| GH12 | 0.002379 | 0.003212 | 9 | 27 | 0 | 0 | 0 | 2 |
| GH28 | 0.022888 | 0.007116 | 148 | 99 | 244 | 6 | 6 | 10 |
| GH53 | 0.006384 | 0.008374 | 27 | 173 | 1066 | 12 | 0 | 84 |
| Total | 0.031923 | 0.020648 | 193 | 317 | 4218 | 78 | 6 | 96 |
| Debranching enzymes | | | | | | | | |
| GH51 | 0.000447 | 0.000966 | 11 | 31 | 0 | 18 | 0 | 0 |
| GH54 | 0 | 0 | 0 | 0 | 111 | 0 | 0 | 0 |
| GH67 | 0.00112 | 0.000283 | 2 | 9 | 1090 | 10 | 0 | 0 |
| GH78 | 0.014254 | 0.025851 | 56 | 186 | 426 | 0 | 17 | 0 |
| Total | 0.015821 | 0.0271 | 69 | 226 | 1627 | 28 | 17 | 0 |
| Oligosaccharide-degrading enzymes | | | | | | | | |
| GH1 | 0.02297 | 0.016402 | 179 | 312 | 331 | 22 | 64 | 73 |
| GH2 | 0.014116 | 0.011384 | 59 | 266 | 942 | 23 | 22 | 18 |
| GH3 | 0.016511 | 0.024893 | 89 | 416 | 5448 | 69 | 45 | 44 |
| GH29 | 0.000257 | 0.001048 | 9 | 36 | 899 | 0 | 13 | 0 |
| GH35 | 0.002552 | 0.001585 | 16 | 32 | 468 | 3 | 3 | 5 |
| GH38 | 0.080862 | 0.001038 | 193 | 36 | 90 | 11 | 0 | 22 |
| GH39 | 0.033362 | 0.026995 | 116 | 394 | 159 | 3 | 2 | 74 |
| GH42 | 0.003146 | 0.001791 | 28 | 47 | 207 | 24 | 14 | 2 |
| GH43 | 0.014536 | 0.012645 | 95 | 292 | 2313 | 16 | 18 | 16 |
| Total | 0.188312 | 0.097781 | 784 | 1831 | 10857 | 171 | 181 | 254 |
| Total GHs | 0.239880 | 0.160102 | 1099 | 2507 | 18990 | 357 | 207 | 400 |

GH: glycoside hydrolase, P. x: *P. xylostella*, B. g: *Bos grunniens*, N. e: *Nasutitermes exitiou*, A. m: *Apis mellifera*. The number next to the name of the species were the references for the data source of the corresponding species. CAZy: carbohydrate-active enzymes (http://www.cazy.org). The different CAZy families were grouped according to the major functional roles. The “community” means the number of genes participated in the lignocellulosic deconstruction discovered in the gut microbial Metagenomic, the “abundance” indicate the relative abundance of the reads assigned to the total carbohydrate-active enzymes that detected in the gut microbiome. Total GHs: the total number or abundance of GHs listed in this table.

**Supplementary Table 10** Catechol biodegradation genes in *S. litura* gut bacteria

| KO ID | Known activities | Abundance | | Community | | Genus |
| --- | --- | --- | --- | --- | --- | --- |
|  |  | Sl-tar | Sl-art | Sl-tar | Sl-art |  |
| K03381 | Catechol 1, 2-dioxygenase | 0.000224 | 0.000434 | 4 | 17 | *Acinetobacter*, *Pseudomonas* |
| K00446 | catechol 2, 3-dioxygenase | 1.38E-07 | 0.000111 | 1 | 4 | *Pseudomonas* |
| K01856 | muconate cycloisomerase | 0.000249 | 0.000301 | 2 | 11 | *Acinetobacter*, *Pseudomonas* |
| K03777 | D-lactate dehydrogenase | 0.00028 | 0.000203 | 1 | 23 | *Enterobacteriaceae*, *Xanthomonadaceae* |
| K03464 | muconolactone D-isomerase | 0.000147 | 0.000108 | 1 | 7 | *Pseudomonas*, *Moraxellaceae,*  *Enterobacteriaceae* |
| K01055 | 3-oxoadipate enol-lactonase | 0.000289 | 0.000255 | 1 | 22 | *Pseudomonas* |
| K02554 | 2-keto-4-pentenoate hydratase | 0.000302 | 0.000201 | 1 | 10 | *Xanthomonadaceae*, *Enterobacteriaceae* |
| K01031 | 3-oxoadipate CoA-transferase | 4.62E-07 | 0.000224 | 1 | 12 | *Alcaligenaceae*, *Enterobacteriaceae* |
| K01032 | 3-oxoadipate CoA-transferase | 0.000237 | 0.000276 | 1 | 15 | *Enterobacteriaceae* |
| K01627 | 2-dehydro-3-deoxyphosphooctonate aldolase | 0.000263 | 0.000343 | 1 | 20 | *Acinetobacter*, *Pseudomonas* |
| K01625 | 4-hydroxy-2-  oxoglutarate aldolase | 0.000363 | 0.000383 | 9 | 28 | *Pseudomonas, Enterococcus* |
| K12660 | 2-dehydro-3-deoxy-L-rhamnonate aldolase | 0.000276 | 4.21E-05 | 1 | 4 | *Enterobacteriaceae* |
| K00632 | acetyl-CoA acyltransferase | 0.000651 | 0.000497 | 5 | 24 | *Staphylococcus*, *Pseudomonas* |
| K07823 | 3-oxoadipyl-CoA thiolase | 8.07E-07 | 0.000249 | 1 | 3 | *Pseudomonas* |
| Total |  | 0.003282 | 0.003627 | 30 | 200 |  |

KO ID: KEGG ID; Known activities: the activities of the enzymes; Abundance: Abundance of the genes in the gut microbiota Metagenomic; Midgut community: the number of genes detected in the Metagenomic of *S. litura* gut bacteria; Genus: the *S. litura* gut bacteria which encode the genes.

**Supplementary Table 11** Amino acid biosynthesis genes in *S. litura* gut bacteria

| KO ID | Pathway | KO name | Known activities | Abundance | | Genus |
| --- | --- | --- | --- | --- | --- | --- |
|  |  |  |  | Sl-tar | Sl-art |  |
| K00765 | His | HisG | ATP phosphoribosyl transferase | 0.000283 | 0.00039 | Enterobacteriaceae, *Pseudomonas* |
| K11755 | His | HisIE | phosphoribosyl-AMP cyclohydrolase | 0.000307 | 0.000109 | *Enterococcus casseliflavus*  *Stenotrophomonas maltophilia*, *Enterococcus* |
| K01523 | His | HisE | phosphoribosyl-ATP pyrophosphohydrolase | 0 | 0.000156 | *Brucellaceae*, *Pseudomonas*, *Burkholderiales* |
| K01814 | His | HisA | phosphoribosylformimino-5-aminoimidazole carboxamide ribotide isomerase | 0.000312 | 0.000335 | *Burkholderiales*, *Enterococcus casseliflavus*,  *Pseudomonas* |
| K02500 | His | HisF | cyclase | 0.000311 | 0.000372 | *Enterococcus casseliflavus*, Enterobacteriaceae,  *Acinetobacter* |
| K01089 | His | HisB | imidazoleglycerol-phosphate dehydratase/ histidinol-phosphatase | 0.000298 | 8.34E-05 | Enterobacteriaceae |
| K00817 | His | HisC | histidinol-phosphate aminotransferase | 0.000686 | 0.000501 | *Sphingobacterium*, Burkholderiales, Xanthomonadaceae |
| K00013 | His | HisD | histidinol dehydrogenase | 0.000386 | 0.000481 | *Actinomycetales*, *Pseudomonas*, *Comamonas*, Enterobacteriaceae |
| K00928 | Thr | LysC | aspartate kinase | 0.000441 | 0.000529 | *Acinetobacter*, *Pseudomonas*,  *Bacillus*, Brucellaceae |
| K00133 | Thr | Asd | aspartate-semialdehyde dehydrogenase | 0.000707 | 0.000718 | Enterobacteriaceae, Xanthomonadaceae |
| K12524 | Thr | ThrA | bifunctional aspartokinase / homoserine dehydrogenase 1 | 0.0003 | 0.000222 | Actinomycetales, Enterobacteriaceae |
| K00872 | Thr | ThrB | homoserine kinase | 0.000387 | 0.000166 | Comamonadaceae, *Enterococcus* |
| K01733 | Thr | ThrC | threonine synthase | 0.000394 | 0.000434 | Enterobacteriaceae |
| K14260 | Ala | AlaA | alanine-synthesizing transaminase | 0.000302 | 0.000331 | *Pseudomonas*, Enterobacteriaceae |
| K00549 | Met | MetE | 5-methyltetrahydropteroyltriglutamate--  homocysteine methyltransferase | 0.000908 | 0.000533 | Burkholderiales, *Pseudomonas*,  *Achromobacter xylosoxidans*, Enterobacteriaceae |
| K01652 | Val, Leu, Ile | ilvH | acetolactate synthase I/II/III large subunit | 0.002011 | 0.001397 | Enterobacteriaceae, *Pseudomonas*, Brucellaceae |
| K00053 | Val, Leu, Ile | ilvC | ketol-acid reductoisomerase | 0.000342 | 0.000338 | *Acinetobacter*, Enterobacteriaceae |
| K01687 | Val, Leu, Ile | ilvD | dihydroxy-acid dehydratase | 0.000385 | 0.001298 | *Pseudomonas*, Enterobacteriaceae, Brucellaceae |
| K00826 | Val, Leu, Ile | ilvE | branched-chain amino acid aminotransferase | 0.000353 | 0.000369 | *Pseudomonas*, Enterococcaceae, Enterobacteriaceae |
| K01649 | Leu | LeuA | 2-isopropylmalate synthase | 0.000656 | 0.000684 | Enterobacteriaceae, Lactobacillales |
| K01703 | Leu | LeuC | 3-isopropylmalate/(R)-2-methylmalate dehydratase large subunit | 0.000344 | 0.00074 | Enterobacteriaceae, Lactobacillales, Pseudomonadaceae |
| K01704 | Leu | LeuD | 3-isopropylmalate | 0.000282 | 0.000471 | Lactobacillales, Enterobacteriaceae |
| K00052 | Leu | LeuB | 3-isopropylmalate dehydrogenase | 0.000367 | 0.000404 | *Serratia*, Pseudomonadaceae |
| K14170 | Tyr, Phe | pheA | chorismate mutase / prephenate dehydratase | 0.000291 | 0.000296 | Enterobacteriaceae, Lactobacillales |
| K04517 | Tyr | tyrA2 | prephenate dehydrogenase | 3.37E-05 | 7.49E-05 | Brucellaceae, Enterobacteriaceae |
| K00832 | Tyr, Phe | tyrB | aromatic-amino-acid transaminase | 0.000565 | 0.000578 | Burkholderiales |
| K01713 | Phe | pheC | cyclohexadienyl dehydratase | 0 | 2.39E-06 | Enterobacteriaceae, *Acinetobacter*, Pseudomonas |
| K01657 | Trp | trpE | anthranilate synthase component I | 0.000333 | 0.00046 | *Enterococcus casseliflavus*, *Acinetobacter*,  *Pseudomonas* |
| K00766 | Trp | trpD | anthranilate phosphoribosyltransferase | 1.89E-05 | 0.000264 | Enterobacteriaceae |
| K13498 | Trp | trpCF | indole-3-glycerol phosphate synthase | 0.000286 | 7.75E-05 | *Enterococcus casseliflavus* |
| K01695 | Trp | trpA | tryptophan synthase alpha chain | 0.000274 | 0.000303 | Enterobacteriaceae |

KO ID: KEGG ID; Pathway: the KEGG pathway of the orthology participated in; KO name: the name of the KEGG orthology; Known activities: the activities of the enzymes; Abundance: Abundance of the gene in the gut bacteria; Midgut community: the number of genes detected in the Metagenomic of *S. litura* gut bacteria; Genus: the *S. litura* gut bacteria which encode the genes.

**References**

**1.** Dai, X., Zhu, Y., Luo, Y., Song, L., Liu, D., Liu, L., Chen, F., Wang, M., Li, J., and Zeng, X. (2012). Metagenomic insights into the fibrolytic microbiome in yak rumen. *PloS one*, 7, e40430.

**2.** Warnecke, F., Luginbuhl, P., Ivanova, N., Ghassemian, M., Richardson, T.H., et al. (2007). Metagenomic and functional analysis of hindgut microbiota of a wood-feeding higher termite. *Nature* 450, 560-565.

**3.** Engel, P., Martinson, V.G., and Moran, N.A. (2012). Functional diversity within the simple gut microbiota of the honey bee. *Proc Natl Acad Sci U S A* 109, 11002-11007.

**4.** Xia, X., Gurr, G.M., Vasseur, L., Zheng, D., Zhong, H., Qin, B., Lin, J., Wang, Y., Song, F., Li, Y., Lin, H., and You, M. (2017). Metagenomic sequencing of diamondback moth gut microbiome unveils key holobiont adaptations for herbivory. *Front Microbiol*, 8**,** 663.
